# Women’s Perspectives on Smoking During Pregnancy and Factors Influencing Their Willingness to Quit Smoking in Pregnancy: A Study From the United Kingdom

**DOI:** 10.7759/cureus.38890

**Published:** 2023-05-11

**Authors:** Simar Kaur, Walburgh Manhungira, Ajesh Sankar, Rupak Sarkar

**Affiliations:** 1 Department of Obstetrics and Gynaecology, Barnsley Hospital National Health Service Foundation Trust, Barnsley, GBR; 2 Department of Women’s Services, Barnsley Hospital National Health Service Foundation Trust, Barnsley, GBR

**Keywords:** quit, willingness, stop smoking services, risks, pregnancy, smoking

## Abstract

Objective

This was a prospective questionnaire-based survey conducted in the Barnsley District of the United Kingdom among antenatal women smoking during pregnancy. The aim of the study was to assess the awareness of women regarding the risks with smoking during pregnancy, study their smoking behavior, their willingness to quit smoking during pregnancy, and the factors that could influence their intention to quit smoking.

Methods

A cohort of antenatal women smoking during pregnancy was surveyed prior to their contact with the maternity Stop Smoking Services. A well-structured, pre-tested, and validated questionnaire was used to assess their awareness regarding risks with smoking during pregnancy and their willingness to quit smoking during pregnancy. Descriptive statistics were used to analyze the results. Binomial logistic regression (univariate and multivariate) was used to identify the factors influencing the women’s willingness to quit smoking during pregnancy.

Results

Among 66 women surveyed, 52 (79%) were multigravida and 14 (21%) were primigravida, with a mean age of 27.4 ± 5.7 years. Most women (68%) were in the first trimester of their pregnancy. Nearly two-thirds of women (64%) had low educational attainment, 53% were unemployed, 68% lived with family members who smoked, and 35% had mental health problems. One-third (33%) of women had an unsuccessful attempt at quitting smoking in the past. Around 44% of women had a low level of nicotine dependence, while 56% had a moderate level of nicotine dependence. More than three-fourths of women (77%) were aware that smoking during pregnancy is harmful for their baby, though most could not report the specific adverse effects. Nearly half of the women (51.5%) were willing to quit smoking during pregnancy with the rationality of having a healthy baby. On multivariate logistic regression analysis, awareness of the women that smoking during pregnancy has ill effects on the baby (adjusted odds ratio (aOR): 46.459, confidence interval (CI): 5.356-402.961, p value <0.001) was found to be the strongest predictor of willingness to quit smoking during pregnancy. Other determinants found to be significantly associated with willingness to quit smoking during pregnancy were unsuccessful quit attempts in the past (aOR: 0.048, CI: 0.007-0.309, p value 0.001) and the absence of any mental health concerns (aOR: 6.097, CI: 1.105-33.647, p value 0.038).

Conclusion

There is considerable room for raising awareness about the risks of smoking during pregnancy and providing effective smoking cessation and relapse prevention interventions in pregnancy. Obstetricians and midwives should actively participate in providing risk-focused information to pregnant women on smoking during pregnancy and support them in smoking cessation. Various factors such as employment status, nicotine dependence, previous failed attempts at quitting smoking, mental health issues, and awareness levels significantly influence the willingness to quit smoking during pregnancy. Hence, there is an imperative need to identify and address the barriers that could affect a woman’s intention to quit smoking during pregnancy.

## Introduction

Smoking has always been a major cause of public health concern at a global level. Although social norms discourage women from smoking during pregnancy, smoking during pregnancy is still a highly prevalent behavior in many countries. A meta-analysis published in 2018 reported a global prevalence of smoking during pregnancy of 1.7%, with a prevalence of 8.1% in the European region and 1.2% in the Southeast Asian region [1].

In England, 10.4% of pregnant women were smokers at the time of birth in 2019-2020. Although the number of women smoking at the time of birth has fallen in the last decade from 14.6% in 2008-2009 to 10.4% in 2019-2020, the rate of decrease has slowed in recent years, with nearly 9.1% of pregnant women smoking at the time of birth in 2021-2022 [2]. In the 2017 Tobacco Control Plan for England, the government set a target of reducing the prevalence of smoking during pregnancy to 6% or less by 2022 [3].

The present study was conducted in Barnsley District of South Yorkshire County in North-Central England where the prevalence of smoking during pregnancy has always been above the national average. Although the prevalence in this region has dropped significantly from a 23% high in 2011-2012 to 13.6% in 2021-2022, it is still much higher than the national target of 6% and the England average of 9.1% [4]. This high prevalence of smoking during pregnancy prompted us to conduct this study to identify ways to address this public health concern.

Smoking during pregnancy is the leading modifiable risk factor for poor birth outcomes, which include stillbirths, miscarriages, placental abruption, low birth weight, and preterm births [5]. Smoking during pregnancy also increases the risk of children developing several respiratory conditions, attention and hyperactivity difficulties, learning difficulties, obesity, diabetes, and future smoking in adulthood [5-7]. Maternal smoking during pregnancy and post-natal exposure to tobacco smoke are also major risk factors associated with sudden infant death syndrome (SIDS), commonly known as cot death. It has been reported that babies whose mothers smoke are up to three times more likely to die from sudden infant death compared with babies whose mothers do not smoke [5].

Women who smoke during pregnancy are more likely to be younger, unemployed, have low educational attainment, have a lack of social support, have an increased incidence of mental illness, and live with family members who smoke [8]. Women who live with a smoker are six times more likely to smoke during pregnancy. Women experiencing depression during pregnancy are four times more likely to smoke than other women, and this presents a challenge to maternity smoking cessation services [9].

Evidence has shown that, despite various challenges, more women quit smoking when they are pregnant than at any other time in their lives [10]. While quitting smoking at any stage of pregnancy is beneficial, women who stop smoking before 15 weeks of pregnancy reduce the risk of spontaneous premature birth, and having low birth weight babies, to the same as a non-smoker [11].

National Centre for Smoking Cessation and Training (NCSCT) recommends the three A’s approach (Ask, Advice, Act) or Very Brief Advice (VBA) for delivering smoking cessation interventions in maternity care settings. The emphasis of the three As model is for the maternal health team to ask and document the smoking status of all pregnant women, to provide brief advice to women about the importance of smoking cessation, and to act to support cessation by referring pregnant women who smoke to available Stop Smoking Services, who then provide quit smoking assessment, assistance in the form of behavioral support and nicotine replacement therapy, and follow-up [12].

To date, no study has comprehensively evaluated the perception of English women regarding smoking during pregnancy and the factors determinant of their willingness to quit smoking in pregnancy. Findings from this study will help provide better support to women smoking in pregnancy, which is a highly prevalent public health problem in the United Kingdom.

## Materials and methods

This was an anonymous cross-sectional survey conducted using a well-structured, pre-tested, and validated questionnaire. The survey was conducted over a period of four months from May 2022 till August 2022.

Pregnant women smoking during pregnancy were recruited from antenatal clinics using convenience sampling technique. A total of 66 pregnant women smoking during pregnancy were recruited for the study. These women were recruited from midwife-led antenatal clinics during their booking visit once their history of smoking during pregnancy was confirmed. After conducting the survey, the women were referred to the Stop Smoking Services for counseling and support in smoking cessation. It was ensured that the women were surveyed prior to their contact with the Stop Smoking team to get an unbiased account of their awareness levels. Some women were also recruited at a later gestation from consultant-led clinics. These women had been smoking during pregnancy but had refused to engage with the Stop Smoking Services when referred initially at their booking visit. Women who had quit smoking pre-pregnancy or early in pregnancy before attending the antenatal clinics were excluded from the study.

The participants were provided with brief information about the study, and informed consent was obtained prior to participation. They were asked to participate voluntarily by filling out an anonymous questionnaire on their awareness on smoking during pregnancy and their willingness to quit smoking. The participants were assured that the collected data would be kept confidential, and no personal identifiers were requested. The survey was deemed exempt from ethical approval by the Barnsley Hospital Research Committee.

Questionnaire design

Questionnaire was developed with questions based on demographic characteristics of the woman, smoking habits and nicotine dependence, household smoking, mental health, previous attempts at quitting, awareness of risks with smoking, and willingness to quit smoking during pregnancy. The questionnaire was developed based on literature search. For construction and content validity, it was reviewed by four independent experts: two consultants from the Department of Obstetrics and Gynecology, a Public Health Specialist Midwife, and a Stop Smoking Midwife. The questionnaire was tested for face validity in a pilot study by surveying 10 pregnant women from the antenatal clinic to ascertain that the questions were acceptable and the wording was well understood by the respondents. The questionnaire response time was five minutes.

The study was conducted in three steps. First, women were asked to answer the questions in the questionnaire while in the clinic. Second, the midwife or doctor conducting the survey provided answers to the participants and educated them regarding the risks with smoking during pregnancy. They also made them familiar with the advantages of quitting smoking early in pregnancy, alternatives available, and the availability of smoking cessation services to support them. Third, an opt-out referral was done for these women to the maternity Stop Smoking Services. Following the survey, the responses to the questionnaire were collected and analyzed.

Statistical analysis

Data entry was performed using Microsoft Excel 2010, and data analysis was done using IBM SPSS Statistics for Windows, Version 28.0 (Released 2021; IBM Corp, Armonk, NY, United States). Descriptive statistics in the form of count and percentage were used to evaluate the results. The chi-square test was used to compare categorical variables. The willingness of women to quit smoking during pregnancy was assessed using a dichotomous yes/no scale. Binary (univariate and multivariate) logistic regression analyses were performed to identify the factors determinant of the willingness to quit smoking during pregnancy. The willingness to quit smoking during pregnancy was used as the dependent variable. Statistically significant variables in the univariate analysis were used as independent variables and entered into a multivariate logistic regression analysis. The adjusted odds ratio (aOR) and 95% CI were used to identify the odds of willingness to quit smoking during pregnancy for each significant variable. A p value of ≤0.05 was considered statistically significant in all statistical analyses.

## Results

Basic information

A total of 66 pregnant women who smoked during pregnancy were surveyed. Of these, 52 (79%) were multigravida and 14 (21%) were primigravida. The age range of the participants was 17-40 years with the mean age being 27.4 ± 5.7 years. The majority of women (54%) were in the age group of 25-34 years. There were five (8%) teenage pregnancies in our study cohort. Most of the women (45, 68%) were less than 12 weeks pregnant. Nearly 24% of women were between 12 and 20 weeks of gestation, while only 8% were more than 20 weeks pregnant. Women recruited in their second or third trimesters were the ones who had declined to engage with the smoking cessation services at the time of booking. Most women (42, 64%) had low educational attainment, having studied only up to GCSE (General Certificate of Secondary Education) or less. Only 15% of the recruited women had received a university-level education. More than half of the women were unemployed (35, 53%) (Table 1).

**Table 1:** Demographic characteristics of the study population. GCSE: General Certificate of Secondary Education.

Characteristics	No. of participants (N)	Percentage (%)
Parity		
Primigravida	14	21
Multigravida	52	79
Age (years)		
14-24	19	29
25-34	36	54
>35	11	17
Gestational age		
<12 weeks	45	68
12-20 weeks	16	24
>20 weeks	5	8
Educational status		
GCSE or less	42	64
A level	14	21
University level	10	15
Occupational Status		
Unemployed	35	53
Employed	31	47

Nicotine dependence and factors determinant of smoking behavior

Nicotine dependence is commonly described using the Heaviness of Smoking Index (HSI). HSI consists of two variables from the Fagerstrom Test for Nicotine Dependence: time to first cigarette in the morning and the number of cigarettes smoked per day. Based on these variables, nicotine dependence is categorized into three categories: low (HSI score 0-1), medium (HSI score 2-4), and high dependence (HSI score 5-6) [13]. We recorded these two variables for all women in our study population and calculated the individual HSI score.

In our study cohort, 29 (44%) women had a low level of nicotine dependence with a HSI score of 0-1 and 37 (56%) women had a moderate level of nicotine dependence with a HSI score of 2-4. None of the women had a high level of nicotine dependence. This was possibly because most women had cut down on the number of cigarettes smoked per day after getting pregnant. Nearly 88% of the women were long-term smokers, having smoked for longer than five years. Around 12% had smoked in the past one to five years.

Various factors likely to influence smoking behavior such as partner or household smoking, mental health issues, and previous attempts at quitting were assessed. There is evidence that these factors are determinants of smoking behavior and could present as barriers to quitting smoking [14].

More than two-thirds of the women (45, 68%) lived with a partner or family member who smoked. Twenty-three (35%) women had mental health concerns like anxiety and depression. Nearly half of the women (32, 49%) had one or more successful attempts at quitting smoking in the past, which was defined as a smoke-free period of at least four weeks. Twenty-two women (33%) had an unsuccessful attempt at quitting smoking in the past, while 12 (18%) had never attempted to quit (Table 2).

**Table 2:** Nicotine dependence and factors related to smoking behavior. HSI: Heaviness of Smoking Index.

Variables	Number	Percentage (%)
HSI/nicotine dependence		
0-1	29	44
2-4	37	56
Mental health disorders		
Yes	23	35
No	43	65
Household smoking		
Yes	45	68
No	21	32
Previous quit attempt		
Successful	32	49
Unsuccessful	22	33
Never attempted quitting	12	18

Awareness of women regarding smoking during pregnancy

**Figure 1:**
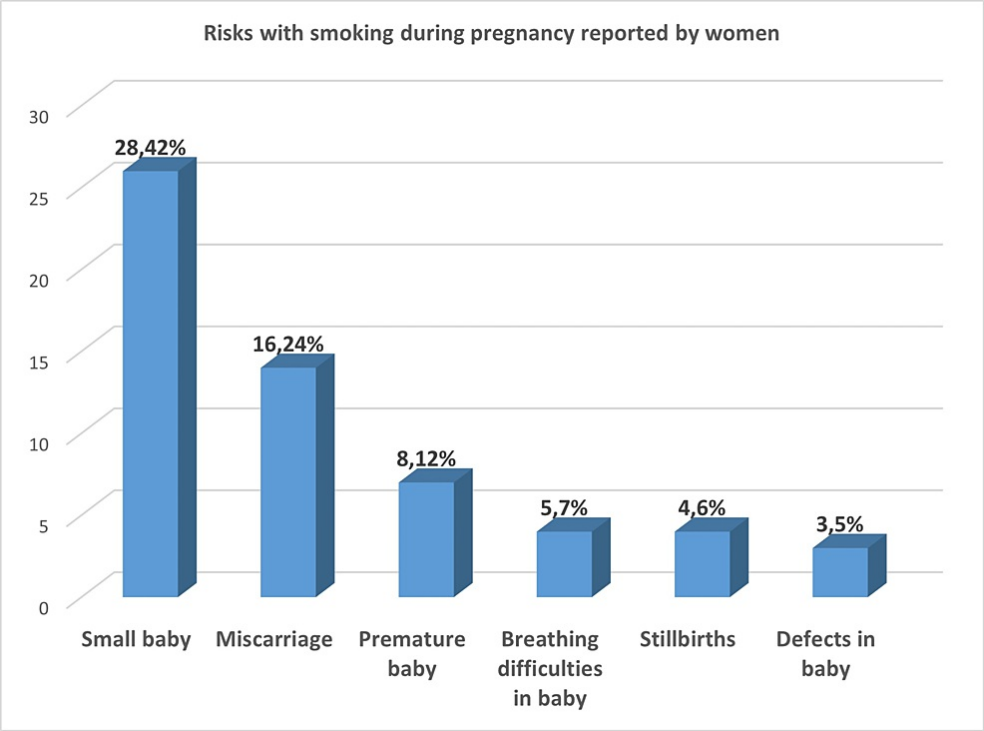
Risks with smoking during pregnancy reported by women.

Nearly 77% of women were aware that smoking during pregnancy is harmful for the baby. These women were asked to specify the risks to the baby due to maternal smoking in a free-form box. Some of the risks associated with smoking during pregnancy that were stated by the women were low-birth-weight baby, premature births, stillbirths, miscarriages, congenital defects, and breathing difficulties in the baby. Risk of a low-birth-weight baby and miscarriage were among the most reported adverse outcomes, reported by 42% and 24% of women, respectively (Figure 1).

A statistically significant association was seen between the educational status of women and their awareness regarding the fact that smoking during pregnancy is harmful for the baby (p value 0.023).

Nearly three-fourths of the women (74%) acknowledged that smoking during pregnancy warrants frequent hospital visits for antenatal care and regular growth scans. Almost 60% of women were aware that reducing the number of cigarettes smoked per day does not have the same positive impact on pregnancy outcomes as quitting completely. Most of the women (66%) were aware that there are alternatives to cigarettes in the form of nicotine replacement therapy that can be used in pregnancy to quit smoking (Table 3).

**Table 3:** Awareness of women regarding smoking during pregnancy.

Questions on awareness regarding smoking during pregnancy	Correct response	Participants with correct response N (%)
Smoking in pregnancy carries risks to the baby	Yes	51 (77)
Women smoking in pregnancy need extra antenatal care/ultrasound scans during the antenatal period	Yes	49 (74)
Reducing the number of cigarettes smoked is not as advantageous as completely quitting smoking	Yes	40 (61)
There are alternatives to cigarettes in the form of nicotine replacement therapy that can be used during pregnancy to quit smoking	Yes	45 (66)

Willingness to quit smoking during pregnancy

Nearly half of the women (34, 51.5%) reported that they were thinking about quitting smoking during pregnancy (willing to quit smoking). Others (32, 48.5%) were not considering quitting.

Women were asked the reasons behind wanting to quit or not quit smoking during pregnancy using a multiple response question. The most common reason reported by those inclined to quit smoking during pregnancy was to improve health outcomes for the baby (94%). Other reasons reported were keeping the home smoke-free before the arrival of the baby (44%), for benefits to their own health (38%), to save money (35%) and for achieving a sense of accomplishment (23.5%).

Women who were not keen to quit smoking during pregnancy (N = 32) reported lack of motivation as the most common reason (62.5%). The second most common reason stated was living with family and friends who smoke (50%). Smoking becomes a shared activity for women who live with smokers. It becomes a part of their couple life and part of their wider friend and family circle. Other reasons stated were worried about withdrawal symptoms (47%) and failure at quitting attempts in the past (22%). Nearly 19% of women reported they were struggling with stress/anxiety or depression and smoking cigarettes was helping them overcome that.

Factors influencing willingness to quit smoking during pregnancy

Univariate regression analysis was performed to study the factors influencing willingness to quit smoking during pregnancy. Among the variables studied, those significantly associated (p value <0.05) with willingness to quit smoking during pregnancy were: employment status (p value 0.015), nicotine dependence (p value 0.046), previous quit attempts (p value 0.002), mental health issues (p value 0.014) and awareness that smoking during pregnancy is harmful for the baby (p value 0.003). Statistically significant association was not observed with other variables (Table 4).

**Table 4:** Univariate logistic regression analysis of factors determinant of willingness to quit smoking during pregnancy. OR: odds ratio; CI: confidence interval; GCSE: General Certificate of Secondary Education; HSI: Heaviness of Smoking Index.

Characteristics	No. of participants (N = 66) Number (%)	Not willing to quit smoking (N = 32) Number (%)	Willing to quit smoking (N = 34) Number (%)	p value	OR	95% CI
Parity						
Primigravida	14	7 (50)	7 (50)	Ref.		
Multigravida	52	25 (48)	27 (52)	0.898	1.080	0.332-3.516
Age (years)						
14-24	19	8 (42)	11 (58)	Ref.		
25-34	36	17 (47)	19 (53)	0.717	0.813	0.265-2.495
>35	11	7 (64)	4 (36)	0.260	0.416	0.090-1.918
Gestational age						
<12 weeks	45	22 (49)	23 (51)	Ref.		
12-20 weeks	16	7 (44)	9 (56)	0.724	1.230	.390 - 2.875
>20 weeks	5	3 (60)	2 (40)	0.639	.638	.097 - 4.188
Educational status						
GCSE or less	42	23 (55)	19 (45)	Ref.		
A level	14	4 (29)	10 (71)	0.097	3.026	0.817-11.206
University level	10	5 (50)	5 (50)	0.786	1.211	0.304-4.814
Employment status						
Unemployed	35	22 (63)	13 (37)	Ref.		
Employed	31	10 (32)	21 (68)	0.015	3.554	1.284-9.840
HSI/nicotine dependence						
0-1	29	10 (34.5)	19 (65.5)	Ref.		
2-4	37	22 (59.5)	15 (40.5)	0.046	0.359	0.131-0.984
Mental health disorders						
Yes	23	16 (70)	7 (30)	Ref.		
No	43	16 (37)	27 (63)	0.014	3.857	1.307-11.383
Household smoking						
Yes	45	24 (53)	21 (47)	Ref.		
No	21	8 (38)	13 (62)	0.251	1.857	0.645-5.348
Previous quit attempts						
Successful	32	9 (28)	23 (72)	Ref.		
Unsuccessful	22	16 (73)	6 (27)	0.002	0.147	0.044-0.494
Never attempted	12	7 (58)	5 (42)	0.071	0.280	0.070-1.114
Awareness that smoking in pregnancy carries risk to the baby						
Unaware	15	13 (87)	2 (13)	Ref.		
Aware	51	19 (37)	32 (63)	0.003	10.947	2.225-53.858

The results of the multivariate logistic regression model are reported in Table 5. Women who were aware that smoking during pregnancy has ill effects on the baby were more likely to quit smoking during pregnancy (p value <0.001; aOR: 46.459, CI: 5.356- 402.961). Likewise, women without any mental illness were more likely to quit smoking during pregnancy (p value 0.038; aOR: 6.097, CI: 1.105-33.647) while those with previous unsuccessful attempts at quitting had lower odds of quitting smoking during pregnancy (p value 0.001, aOR: 0.048, CI: 0.007-0.309). 

**Table 5:** Multivariate logistic regression analysis of factors determinant of willingness to quit smoking during pregnancy. aOR: adjusted odds ratio; CI: confidence interval.

Variable	aOR	95% CI	p value
Awareness that smoking in pregnancy is harmful for the baby	46.459	5.356-402.961	<0.001
Previous unsuccessful quit attempts	0.048	0.007-0.309	0.001
Absence of mental health issues	6.097	1.105-33.647	0.038

## Discussion

In this study, the majority of women (77%) who smoked during pregnancy were aware that smoking during pregnancy is harmful to the baby, but their awareness about specific risks to the baby like stillbirths, congenital defects, and preterm births was low, ranging from 5% to 42%. These findings were in line with a similar study conducted in the United States, where participants had a low-to-moderate awareness (9%-57%) of most adverse outcomes with smoking during pregnancy [15]. Another pilot study conducted in the United States also reported low awareness among pregnant women regarding the risk of long-term respiratory morbidity associated with maternal smoking in their offspring [16]. A similar study from the central part of India reported a very low overall level of awareness (12.3%) about the adverse effects caused by the use of tobacco during pregnancy [17].

Nearly half of the pregnant women (51.5%) were inclined towards quitting smoking during pregnancy primarily to improve the health outcome of their baby. Women who were aware that smoking during pregnancy is harmful for the baby had a higher likelihood of quitting smoking during pregnancy (aOR: 46.459, CI: 5.356-402.961). Clearly, if women smoking during pregnancy are given adequate information about the risks associated with smoking in pregnancy by their healthcare providers, that will go a long way in motivating them to quit smoking during pregnancy.

All maternity healthcare professionals, especially the obstetricians and midwives who are the first point of contact for pregnant women, must be prepared to support them with smoking cessation. Healthcare providers should give an in-depth explanation of the risks with smoking during pregnancy, explain the need for additional fetal growth monitoring, and discuss with women the timing of smoking cessation during pregnancy (though, ideally, before pregnancy or by 15 weeks of pregnancy but is beneficial at any time in pregnancy).

A Cochrane review illustrated that psychosocial interventions like providing information on fetal health status could decrease the number of women who smoke in late pregnancy by 35% [8]. This could include simple interventions like highlighting the reduced growth velocity of the fetus on growth charts.

Women must also be apprised of the risks with smoking during pregnancy by displaying an audio-visual guide in antenatal clinics and ultrasound waiting areas and sending out information leaflets. A study conducted in the United States reported that the use of strong graphics or photographs depicting risks to babies is more likely to generate strong reactions among women and motivate them to quit smoking during pregnancy [15].

Nearly 40% of our women believed that cutting down on the number of cigarettes in pregnancy was enough to reduce the adverse effects of smoking on the baby. There is little evidence to suggest that reducing cigarette consumption has a positive effect on smoking-related outcomes. This is most likely because smokers who cut down compensate for this reduction by increasing the intensity with which they smoke cigarettes to maintain a relatively stable level of nicotine intake. This concept of compensatory smoking should be explained to all women during their antenatal visits, especially when women report they have cut down on the number of cigarettes but have not yet achieved complete abstinence. [18].

Our study cohort majorly consisted of women, with low educational status with nearly half of them being unemployed. More than two-thirds of women (68%) lived with a partner who smoked, more than one third (35%) had mental health concerns, and more than half (56%) had a moderate level of nicotine dependence. The presence of these social and demographic factors has been reported to influence a woman’s smoking status [8]. Given the presence of these factors, there is an imperative need to address them to help women quit smoking during pregnancy, improve pregnancy outcomes, give a smoke-free start to every baby, and improve the health of the women and their partners.

On logistic regression analysis, the absence of mental illness was identified as a significant determinant for willingness to quit smoking during pregnancy in our study (aOR: 6.097, CI: 1.105-33.647). Women struggling with stress, anxiety, and depression often perceive smoking as a way of coping with their symptoms. They also perceive quitting to be more difficult compared to those without stress or anxiety. These women should be offered effective mental health support from perinatal mental health team in addition to the stop smoking support to facilitate smoking cessation. 

In our study, household smoking was not found to be significantly associated with willingness to quit smoking during pregnancy. A similar study from India reported tobacco consumption by husband (aOR: 36.16, CI: 22.89-68.86), and a female friend (aOR: 22.29, CI: 13.11-31.82) to be the strongest predictors of tobacco consumption by pregnant women [17]. Women who live with smokers are exposed to more positive norms and cues towards smoking, which can make quitting more difficult. It is imperative to assess the status of household smoking in antenatal clinics and encourage the partner or family members to quit smoking.

Multivariate logistic regression analysis showed a statistically significant association between unsuccessful attempts at quitting smoking in the past and willingness to quit smoking during pregnancy (aOR: 0.048, CI: 0.007-0.309). Women with unsuccessful quit attempts in the past develop a fear of failure. It is important to understand that most smokers try to quit a number of times before they are able to quit for good. Equally important to understand is that using willpower alone is the least effective method of quitting smoking. Evidence says that women have three times higher chance of success at quitting smoking with the help of behavioral support and nicotine replacement therapy compared to an unassisted quit attempt [19]. Hence, all women smoking during pregnancy should be timely referred to Stop Smoking Services.

Conversations surrounding smoking cessation in pregnancy are often seen as sensitive and difficult and may harm the bond developing between healthcare providers and the women. The use of non-confrontational brief intervention-based methods may aid health professionals by creating a positive image of behavior change [20]. The healthcare providers must encourage and appreciate pregnant women who successfully quit smoking and continue to abstain from it. The obstetricians and midwives should be offered training on ways to support women on smoking cessation and directed to e-learning resources on smoking cessation in pregnancy like the ones offered by the NCSCT [21].

In the United Kingdom, there are maternity smoking cessation services in most hospitals to support pregnant women with smoking cessation. Every woman identified to be smoking during pregnancy or having a carbon monoxide (CO) reading greater than 4 ppm at the booking visit is given a brief advice on smoking cessation by the midwife and is referred to the Stop Smoking team [22]. The Stop Smoking team contacts these women within 48 hours of being referred and encourages them to engage with them. Women are provided information on the adverse effects of smoking on pregnancy outcomes and are offered behavioral and pharmacological support for smoking cessation. Smoking cessation support is also offered to the partners or family members, as their smoking status can substantially influence the woman’s likelihood of achieving abstinence from smoking. The women are sometimes also offered financial incentives to keep them motivated.

Despite widely accepted health risks and available support, smoking during pregnancy remains a public health issue in the United Kingdom, and smoking cessation remains a key health promotion topic for maternity healthcare professionals. Though the services offered by the Stop Smoking team are paramount, the role of obstetricians and midwives in identifying a woman smoking during pregnancy at first contact, encouraging her to quit smoking, and referring her to the Stop Smoking team remains of chief importance.

The limitation of our study was the small sample size. By accessing a larger population, more accurate information could be obtained on women’s perspectives on smoking during pregnancy and the factors that could influence their attempts at smoking cessation. Another limitation was the convenient method of sampling used, which is a non-probability sampling strategy. Hence, the sample subset may not be truly representative of the general population. The information obtained on the attitudes and opinions of women from a multicenter survey would be more representative of the entire population. 

## Conclusions

There is considerable room for raising awareness among women about the risks of smoking during pregnancy. Various factors such as employment status, level of nicotine dependence, previous failed attempts at quitting smoking, presence of mental health issues, and level of awareness significantly influence the willingness of a woman to quit smoking during pregnancy. Hence, there is an imperative need to identify and address the barriers that could affect a woman’s intention to quit smoking during pregnancy.

Active involvement by obstetricians and midwives is essential in providing awareness regarding the risks with smoking during pregnancy and helping women in overcoming the barriers in smoking cessation. Pregnancy is a motivational trigger for most women to change or adopt a new behavior. Hence, a pregnant woman is highly likely to engage with smoking cessation services to quit smoking if encouraged to do so by the primary maternity healthcare provider. Encouraging women to quit smoking pre-pregnancy or early in pregnancy will go a long way in reducing the risk of stillbirths and pave a healthy and smoke-free start for every baby.
